# FP-MAP: an extensive library of fingerprint-based molecular activity prediction tools

**DOI:** 10.3389/fchem.2023.1239467

**Published:** 2023-08-15

**Authors:** Vishwesh Venkatraman

**Affiliations:** Department of Chemistry, Norwegian University of Science and Technology, Trondheim, Norway

**Keywords:** fingerprints, random forests, neglected diseases, classification, regression, graph neural networks

## Abstract

Discovering new drugs for disease treatment is challenging, requiring a multidisciplinary effort as well as time, and resources. With a view to improving hit discovery and lead compound identification, machine learning (ML) approaches are being increasingly used in the decision-making process. Although a number of ML-based studies have been published, most studies only report fragments of the wider range of bioactivities wherein each model typically focuses on a particular disease. This study introduces FP-MAP, an extensive atlas of fingerprint-based prediction models that covers a diverse range of activities including neglected tropical diseases (caused by viral, bacterial and parasitic pathogens) as well as other targets implicated in diseases such as Alzheimer’s. To arrive at the best predictive models, performance of ≈4,000 classification/regression models were evaluated on different bioactivity data sets using 12 different molecular fingerprints. The best performing models that achieved test set AUC values of 0.62–0.99 have been integrated into an easy-to-use graphical user interface that can be downloaded from https://gitlab.com/vishsoft/fpmap.

## 1 Introduction

Development of therapeutic drugs is an expensive affair with expected costs ranging from $1 billion to more than $2 billion (Schlander et al., 2021) depending on the therapeutic area and disease complexity. The molecular universe is very large with some estimates placing their number at over 10^60^ different drug-like molecules (Reymond and Awale, 2012). There now exist virtual databases such as SAVI (Patel et al., 2020), ZINC (Irwin et al., 2020), ENAMINE (Sadybekov et al., 2021) and the GDB (Reymond and Awale, 2012), that contain hundreds-of-millions to billions of diverse molecules that can be queried to find novel molecules of interest. Since making and testing all the interesting compounds is out of question, there is a need to weed out molecules that are not relevant to drug discovery, i.e., exclude those that exhibit less than acceptable biological activity. However, despite recent efforts (Gorgulla et al., 2020; Bender et al., 2021; Glaser et al., 2021; Gentile et al., 2022; Luttens et al., 2022) reliable simulation methods for large scale activity prediction still remain elusive.

To circumvent some of the challenges, machine learning (ML) approaches are being increasingly used for the prediction of biological activities (Cova and Pais, 2019; Lane et al., 2020; Elbadawi et al., 2021). Here, a wide variety of ML algorithms are trained to identify quantitative structure-activity relationships (Wu et al., 2020; Pillai et al., 2022) that are then used to generate predictions that are subsequently used to select the next screening subset, thereby facilitating more efficient use of time and resources (Dreiman et al., 2021; Graff et al., 2021). Key to the success of the models is the quality and amount of data, the molecular representation and the ML method. Although annotated data remains limited, public databases such as ChemBL (Gaulton et al., 2016) and concerted efforts to make data open access (Capuzzi et al., 2017; Wu et al., 2018; Kexin Huang, 2020) have spawned a number of machine learning projects (Mayr et al., 2018; Lane et al., 2020). Molecular representation plays a crucial role in machine learning and is problem-specific (David et al., 2020; Raghunathan and Priyakumar, 2021) with popular choices being fingerprints (bit string indicating absence/presence of features), molecular graphs (network of nodes and edges) and molecular embeddings (Jaeger et al., 2018). While a wide array of ML algorithms have been employed, there is no clear winner, although ensemble learning has been shown to yield good results across many data sets (Wu et al., 2020; Sabando et al., 2021).

To help researchers ease their way into drug discovery and carry out screening experiments, automated ML platforms and web-based tools have gained significant traction in recent years (Liu et al., 2019; Singh et al., 2020; Togo et al., 2022). While a great number of software and web tools are devoted to physicochemical properties, ADMET and ADMET-related filtering (Venkatraman, 2021; Xiong et al., 2021), prediction software that cover a broad range of biological activities are relatively fewer (Scotti et al., 2022). In many cases, the prediction software are limited to a single disease or class and largely operate as online prediction services that are not easily amenable to large scale screening (see Table 1 for a short summary of recently published software tools that provide online prediction services). Furthermore, in spite of a large number of published models, only a few are publicly accessible while many are part of proprietary collections (Ma et al., 2015; Aleksić et al., 2021). Cheminformatics web services and software for bioactivity prediction is indeed growing (Ruusmann et al., 2015) and a great many software and services such as VCCLab (Tetko et al., 2005) and DPubchem (Soufan et al., 2018) offer a platform for calculations of a comprehensive series of molecular properties and data analysis. Other services such as AssayCentral (www.collaborationspharma.com/assay-central) focus on allowing pharmaceuticals or individuals to leverage their internal databases. In a recent study, over 5,000 machine learning models built from data sets extracted from ChemBL have been made available on the AssayCentral platform (Lane et al., 2020).

**TABLE 1:** Table lists several open access software for drug activity prediction.

Software	Description	Distribution
HergSPred (Zhang et al., 2022b)	hERG Blockers/Nonblocker	Web
MolPredictX (Scotti et al., 2022)	predictions for 27 diseases	Web
mycoCSM (Pires and Ascher, 2020)	screen hits against Mycobacteria	Web
pdCSM-PPI (Rodrigues et al., 2021)	Protein-Protein Interaction Inhibitors	Web
pdCSM-GPCR (Velloso et al., 2021)	GPCR inhibitors	Web
cardioToxCSM (Iftkhar et al., 2022)	Cardiotoxicity	Web
pdCSM-cancer (Al-Jarf et al., 2021)	Cancer drugs	Web
ChemBC (He et al., 2021)	Breast Cancer	Web/Standalone
ChemTB (Ye et al., 2021)	*Mycobacterium tuberculosis*	Web
MAIP (Bosc et al., 2021)	blood-stage malaria inhibitors	Web
S2DV (Shao et al., 2022)	anti-hepatitis B drug screening	Web
HRGCN (Wu et al., 2021a)	Toxicity, HIV and BACE inhibitor	Web
MolPMoFiT (Tinivella et al., 2021)	HIV and BBB penetration	Standalone
HIVprotI (Qureshi et al., 2018)	HIV protein inhibitors	Web
EBOLApred (Adams et al., 2022)	Ebola virus cell entry inhibitors	Web
embryoTox (Aljarf et al., 2023)	Teratogenicity of Small Molecules	Web
InflamNat (Zhang et al., 2022a)	anti-inflammatory drug screening	Web

This article presents FP-MAP, a fast fingerprint-based bioactivity prediction tool to help identify active molecules for a number of pharmaceutically relevant targets. In particular FP-MAP sets out to assemble predictive models for diseases and targets for which there are currently no publicly available software. In order to build the models, 12 different fingerprints were trialled and the best-performing models (based on 5-fold cross-validated statistics) were retained. A pre-assessment step was carried out wherein the predictive ability of the fingerprint models was found to be comparable or an improvement over previously reported results for multiple data sets. For the different classification models computed for severely imbalanced data sets, moderate to high area under the ROC curve (AUC) values of 0.61–0.95 were obtained. FP-MAP currently offers 24 different classification models for rapid screening of compounds against a number of diseases caused by bacteria and parasites such as schistosomiasis, cholera and malaria as well as other targets implicated in diseases such as Alzheimer’s, cancer and cardiomyopathy. To facilitate the use of the models, the software has been made available as an easy to use graphical user interface and can be accessed from https://gitlab.com/vishsoft/fpmap.

## 2 Materials and methods

### 2.1 Data sets studied

In order to assess the predictive ability of the fingerprint-based machine learning models, multiple data set were analysed. A set of 79 pharmacologically important biological targets were initially used as a means to benchmark performance, proceeding which model performance was assessed on more challenging targets that are described briefly in the following sections.

#### 2.1.1 Chemical toxicology

The toxicology data set includes 79 pharmacologically important biological targets (see Supplementary Table S1 in the SI). The compounds were extracted from ChemBL and ToxCast and were categorized as binders if the reported activities against the human protein targets (K_*i*_/K_*d*_/IC_50_/EC_50_) were ≤10 *μ*M and as non-binders if activities were > 10 *μ*M (Allen et al., 2020). For the data sets, deep learning neural networks yielded test data accuracies of 92% ± 4%.

#### 2.1.2 ExcapeDB

The ExcapeDB (Sun et al., 2017) database comprises activity data of chemical compounds on an array of protein targets. The data were extracted from publicly available databases such as PubChem and ChEMBL. A set of 12 gene targets were evaluated in this study.

#### 2.1.3 PubChem

An important source of data is the PubChem Bioassay (Kim et al., 2022) which contains small-molecule screening data. This study analyses multiple data sets drawn from the PubChem archive where the focus is primarily on rare diseases related to genetic disorders and neglected tropical diseases.

##### 2.1.3.1 Bubonic plague

YopH (*Yersinia* outer protein H) is a protein essential for the virulence of *yersinia pestis* (Bubonic plague). The data set consists of ∼140,000 compounds that were part of a high throughput screening assay (https://pubchem.ncbi.nlm.nih.gov/bioassay/898) to identify compounds that can interfere with YopH functionality. Actives were defined as those with inhibition ≥50%.

##### 2.1.3.2 Potassium channel blockers

The KCNQ1 (Potassium Voltage-Gated Channel Subfamily Q Member 1) gene codes for the potassium channel protein which is critical for electrical signaling in cells. In an effort to identify compounds that inhibit KCNQ1 potassium channels, a little over 300,000 compounds were assayed (https://pubchem.ncbi.nlm.nih.gov/bioassay/2642).

##### 2.1.3.3 Trypanosoma brucei hexokinase

*Trypanosoma brucei* is a protozoan parasite that causes African sleeping sickness. Glucose metabolism is essential for the parasite, and hexokinases have been considered as important therapeutic targets. The data set consists of a little over 220,000 compounds (https://pubchem.ncbi.nlm.nih.gov/bioassay/1430) where the goal was to identify specific inhibitors of *Trypanosoma brucei* hexokinase activity (Morris et al., 2006). Compounds with more than 50% inhibition are considered to be active.

##### 2.1.3.4 Antimalarials

The MMV St. Jude malaria data set (Verras et al., 2017) contains a set of 305,810 compounds that were assayed for malaria blood stage inhibitory activity.

##### 2.1.3.5 Leishmania

Leishmaniasis is a neglected disease caused by protozoan parasites. Currently no safe vaccines exist. The data set earlier studied by Casanova-Alvarez et al. (2021), includes ∼ 196,000 compounds that have been tested for leishmania parasite growth and viability inhibition against *Leishmania major* promastigotes.

##### 2.1.3.6 Activators of kallikrein-7

The chymotrypsin-like serine protease kallikrein-7 (K7) zymogen has been shown to play critical roles in skin diseases and tumour progression. K7 expression was significantly decreased in the brains of Alzheimer’s disease (AD) patients (Kidana et al., 2018). Compounds that can directly activate K7 without a requirement for proteolytic processing can enable development of new therapeutics for cancer, skin diseases, and AD. The data set contains over 350,000 compounds (https://pubchem.ncbi.nlm.nih.gov/bioassay/652039).

##### 2.1.3.7 Dengue

Antiviral drugs against dengue infection are much needed with an estimated 4 billion people living in areas with a risk of dengue (https://www.who.int/news-room/fact-sheets/detail/dengue-and-severe-dengue). The data set consists of over 10,000 compounds (https://pubchem.ncbi.nlm.nih.gov/bioassay/540333) wherein active compounds showed inhibition of cytopathic effect-based assay greater than 13.25%.

##### 2.1.3.8 VIM2 inhibitors

Antibiotic resistance caused by *β*-lactamase production presents significant challenges to the efficacy of *β*-lactam antibiotics. Given the paucity of new antibiotics, high throughput screening assay to identify inhibitors of the Verona Integron-Encoded Metallo-*β*-Lactamase 2 (VIM-2) have been carried out.

##### 2.1.3.9 Cholera

Cholera is acute diarrhoeal disease caused by infection of the intestine with *Vibrio cholerae* bacteria. Due to the prevalence of multi-drug resistance in these bacteria new drugs to combat these pathogens are required. The data set contains over 130,000 compounds (https://pubchem.ncbi.nlm.nih.gov/bioassay/504770) of which 350 compounds showed potent cidal activity against *V. cholerae*.

##### 2.1.3.10 Schistosomiasis

Caused by parasitic worms (such as Schistosoma mansoni), Schistosomiasis is prevalent in tropical and subtropical areas particularly among poor and rural communities with ≈90% of those requiring treatment living in Africa (https://www.who.int/news-room/fact-sheets/detail/schistosomiasis). Owing to the parasite becoming drug resistant and lack of suitable alternative therapies, new targets and drugs for schistosomiasis treatment are foremost importance. The data set contains over 300,000 compounds tested for inhibition of Thioredoxin glutathione reductase (https://pubchem.ncbi.nlm.nih.gov/bioassay/485364). Compounds defined as inconclusive were excluded from further analysis.

##### 2.1.3.11 Glucocerebrosidase

The deficiency of *β*-glucocerebrosidase results in Gaucher disease, a rare genetic disorder for which there is no cure but can be controlled using drugs. The PubChem assay (https://pubchem.ncbi.nlm.nih.gov/bioassay/360) screens for small molecule inhibitors that could potentially act as molecular chaperones on the mutant forms *β*-glucocerebrosidase.

##### 2.1.3.12 Leishmania

Available leishmaniasis treatments are limited and increasingly confronted by issues such as toxic side effects and chemoresistance. The data set includes close to 200,000 compounds assayed for Leishmania parasite growth inhibition https://pubchem.ncbi.nlm.nih.gov/bioassay/1063.

### 2.2 Molecular fingerprint representations

Molecular fingerprints have a long history of having been used in similarity searching (Muegge and Hu, 2022). Their popularity can be largely attributed to their ability to evaluate vast libraries of compounds using just a fraction of the resources and time (Venkatraman et al., 2022) that would otherwise be used with more compute intensive approaches. The fingerprint representations used in this study can be grouped into:

1. Those based on pre-defined generic substructures/keys (Bender et al., 2009) such as PUBCHEM (NCBI, 2009), Klekota-Roth (Klekota and Roth, 2008) and MACCS (Durant et al., 2002)
2. Circular topological fingerprints (Rogers and Hahn, 2010) that represent molecular structures using circular atom neighborhoods (defined by a radius). The extended connectivity fingerprints (ECFP) and feature-class fingerprints belongs to this group.
3. Topological path-based fingerprints in which linear/branched paths up to a certain length are enumerated and encoded. Here, RDKit topological fingerprints (Landrum, 2022) of path sizes 5, 6, and 7 bonds have been used.

Table 2 provides a summary of the fingerprints used for predictive modelling. Machine learning models for a total of 12 different fingerprints adapted from a set of fingerprints studied earlier by Riniker and Landrum (2013) were evaluated. These fingerprints have been widely used as molecular representations with applications in similarity searching and modelling structure-activity relationships (Zagidullin et al., 2021; Muegge and Hu, 2022; Orosz et al., 2022). The fingerprints were generated using available routines in open source cheminformatics software such as RDKit (Landrum, 2022) and the Chemistry Development Kit (Willighagen et al., 2017).

**TABLE 2:** Molecular fingerprints used for predictive modelling.

Fingerprint	Group	Size (bits)
ECFP2 Rogers and Hahn (2010); Willighagen et al. (2017)	Circular	1,024
ECFP4 (Rogers and Hahn, 2010; Willighagen et al., 2017)	Circular	1,024
ECFP6 (Rogers and Hahn, 2010; Willighagen et al., 2017)	Circular	1,024
FCFP2 (Rogers and Hahn, 2010; Willighagen et al., 2017)	Circular	1,024
FCFP4 (Rogers and Hahn, 2010; Willighagen et al., 2017)	Circular	1,024
FCFP6 (Rogers and Hahn, 2010; Willighagen et al., 2017)	Circular	1,024
MACCS (Durant et al., 2002)	Substructure	166
PUBCHEM (NCBI, 2009; Willighagen et al., 2017)	Substructure	881
AVALON (Landrum, 2022)	Substructure	1,024
RDK5 (Landrum, 2022)	Path	1,024
RDK6 (Landrum, 2022)	Path	1,024
RDK7 (Landrum, 2022)	Path	1,024

For the extended connectivity fingerprints (ECFP) and functional class fingerprints (FCFP), the values of 2, 4, and 6 indicate the diameters of the atom neighbourhoods. For RDKit fingerprints the values of 5, 6, and 7 indicate the size (in bonds) of the paths considered.

### 2.3 Modelling

Prior to modelling, a data cleaning step was followed wherein the SMILES were standardized and cleaned using MayaChemTools (Sud, 2016). Subsequently, for each data set, the available data was randomly split into calibration (80%) and test sets (20%). Model training was performed using random forests (Breiman, 2001) (RF) where the number of trees was set to 500. A 5-fold cross-validation on the training set was carried out to tune the parameter “mtry” (number of input features that will be randomly sampled at each split when creating the tree models). Prediction performances were subsequently assessed on the test set. The train/test splitting (80:20 ratio) was repeated 3 times to assess variability of the prediction performance and to rule out any significant impact on performance owing to selection. The RF models were built using the *caret* (Kuhn, 2022) and *ranger* (Wright and Ziegler, 2017) packages in R (R Core Team, 2022). The classification models were evaluated using the balanced accuracy score (Kelleher et al., 2015) which accounts for the skewness of the class distributions

$$BACC=\frac{Sensitivity+Specificity}{2}$$ (1)

Here, the sensitivity $\left(\frac{TP}{TP+FN}\right)$ and specificity $\left(\frac{TN}{TN+FP}\right)$ are defined in terms of the counts of true positive (TP), true negative (TN), false positive (FP) and false negative (FN). For comparison, other metrics such as the area under the curve (AUC) are also reported.

In order to address the issue of applicability domain of the models, outlier detection using isolation forest (Liu et al., 2008) has been employed. Here, a test compound is assessed for its tendency to separate from the majority of samples using an isolation forest constructed from binary trees. Isolation forests make use of decision tree (are an unsupervised version of random forests) and work on the assumption that for non-outlier points, it takes a large number of splits to separate them into individual buckets (i.e., number of partitions that it takes to isolate a point). By contrast, anomalous points are likely to take much shorter paths for isolation. In this study, the isofor package in R was used to identify potential outliers.

## 3 Results and discussion

### 3.1 Performance benchmarking

**FIGURE 1:**
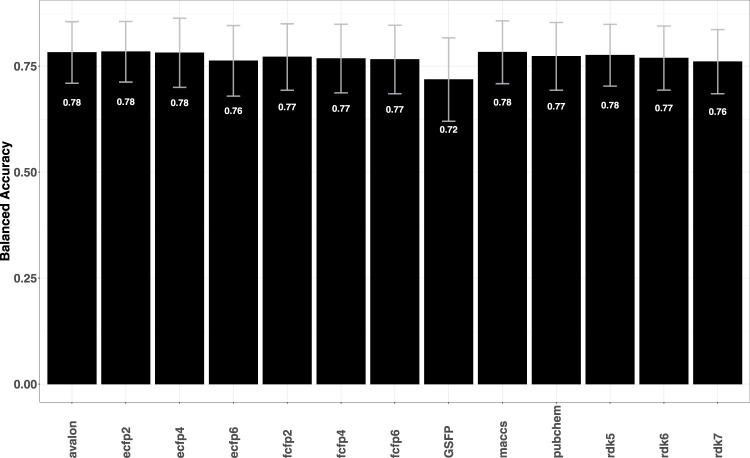
Plot shows the average AUC values for each fingerprint model averaged over 6 cardiac toxicity related outcomes. Error bars indicate the variability (standard deviation) of the obtained AUCs. Individual prediction performances of the models can be seen in Supplementary Figuree S3 in the SI.

The performance of the fingerprint models was first assessed on the 79 targets (data summary in Supplementary Table S1 in SI) earlier studied by Allen et al. (2020). The heatmap of the balanced accuracies in Supplementary Figure S1 in the SI shows that with the exception of some selected targets such as MAPK1, PTPN11 and hERG, the fingerprint models perform quite well with average accuracies (average of the BACC values across all targets) close to 0.90 for most targets (see Supplementary Figure S2 in the SI). The prediction results for the fingerprint models compare favourably with the metrics reported for deep learning neural networks (Allen et al., 2020) and can be attributed to the fact that the data sets are relatively balanced (positive data percentage of ≈50%). The fingerprint models were also evaluated against six types of cardiac toxicity outcomes: arrhythmia, cardiac failure, heart block, hERG toxicity, hypertension, and myocardial infarction (see Supplementary Table S1 in the SI). These data sets were previously studied by Iftkhar et al. (2022) who used a combination of graph-based signatures and fingerprints to identify models capable of identifying molecules likely to be toxic. Figure 1 summarizes the performance of the fingerprint models which as can be seen, achieve relatively better predictive performance in terms of the AUC.

**FIGURE 2:**
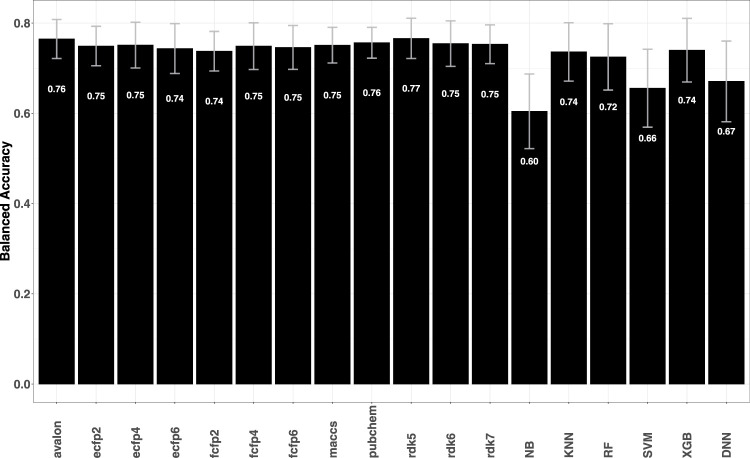
Plot shows the average BACC values for each fingerprint model averaged over 14 breast cancer cell lines. Error bars indicate the variability (standard deviation) of the obtained accuracies. A target-wise summary of the prediction performances of the models can be seen in Supplementary Figure S4 in the SI.

As further validation of the fingerprint models, predictive performance on a series of structurally diverse datasets consisting of 33,757 active and 21,152 inactive compounds for different breast cancer cell lines was also evaluated. The data sets were earlier studied by He et al. (2021), where a number of descriptor-based machine learning models such as naïve Bayes (NB), support vector machine (SVM), *k*-nearest Neighbors (KNN), extreme gradient boosting (XGB) as well as deep learning methods were tested. Comparison of the metrics obtained for fingerprint models with those reported by He et al. (2021) shows that the former achieve higher predictive accuracies with BACC >0.70 (see Figure 2).

Overall, the performance on multiple data sets clearly shows that fingerprints have good predictive power. The majority of the data however, has minimal skew, i.e., near equal numbers of actives and inactives with some even displaying greater bias towards active compounds. Most machine learning approaches are likely to yield strong performances for such balanced data distributions. Data sets drawn from PubChem have typically strongly imbalance and the question is whether fingerprints can yield robust structure–activity relationship models for such data.

### 3.2 Performance evaluation of selected bioactivity data sets

**FIGURE 3:**
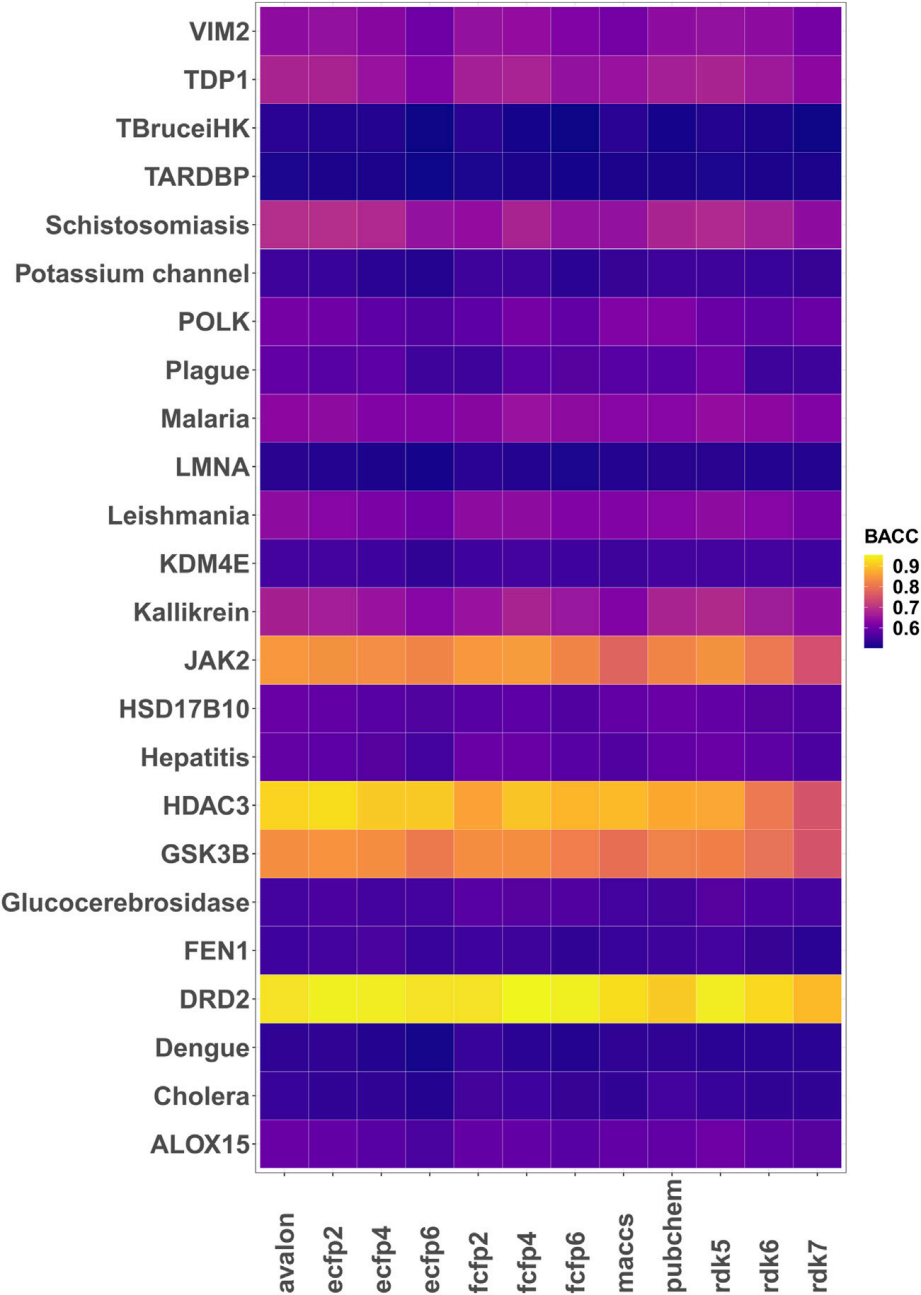
Heatmap of the 5-fold cross validated balanced accuracies (mean of 3 runs) achieved by the different fingerprint models.

Encouraged by the performance of the fingerprints on the different targets, model performance was further assessed on 24 different bioactivity data sets. Table 3 lists the balanced accuracies for the calibration/test sets (average of 3 independent trials) obtained for the targets. Although the performance varies, it is generally seen that the fingerprint models yield reasonable results even for cases with severe imbalance. The heatmap in Figure 3 shows that in a number of cases such as potassium channel inhibitors, KDM4E, LMNA and TARDBP, the selected fingerprints show only a marginal difference in performance with balanced accuracies ≈0.55. Among the fingerprints evaluated in this study, best results were frequently seen to perform well include AVALON, ECFP2/FCFP4/FCFP6 and RDK5.

**TABLE 3:** Table summarizes the random forest classification performance for the various data sets studied.

Disease/Target	Source	#Active/#Inactive	Fingerprint	BACC (Cal/Val)
Malaria	St Jude	2507/303303	FCFP6	0.636/0.640
Kallikrein-7 activator	PubChem^a^	3324/365562	RDK5	0.683/0.689
Hepatitis	PubChem^b^	8443/200362	FCFP4	0.594/0.605
VIM2 Inhibitor	PubChem^c^	2575/288127	FCFP4	0.646/0.648
Leishmania	PubChem^d^	17630/178543	FCFP4	0.638/0.647
Schistosomiasis	PubChem^e^	10701/331424	RDK5	0.686/0.706
Potassium Channels	PubChem^f^	3878/301707	RDK5	0.547/0.550
T.Brucei Hexo Kinase	PubChem^g^	239/220096	AVALON	0.536/0.521
Bubonic Plague	PubChem^h^	223/139693	RDK5	0.598/0.572
*Vibrio cholerae*	PubChem^i^	350/132090	PUBCHEM	0.557/0.578
Dengue	PubChem^j^	318/9920	AVALON	0.532/0.540
Glucocerebrosidase	PubChem^k^	549/45729	FCFP4	0.571/0.547
HSD17B10	ExcapeDB	3408/11510	AVALON	0.592/0.593
KDM4E	ExcapeDB	3938/35058	FCFP4	0.553/0.552
TARDBP	ExcapeDB	12128/387760	RDK5	0.518/0.510
TDP1	ExcapeDB	23083/276558	AVALON	0.679/0.692
DRD2	ExcapeDB	8323/343206	ECFP2	0.947/0.949
FEN1	ExcapeDB	1041/381446	AVALON	0.556/0.548
GSK3B	ExcapeDB	3268/300183	ECFP2	0.843/0.833
HDAC3	ExcapeDB	354/311367	ECFP2	0.864/0.900
JAK2	ExcapeDB	2135/213875	FCFP6	0.851/0.866
LMNA	ExcapeDB	14742/171388	AVALON	0.525/0.515
POLK	ExcapeDB	823/392317	MACCS	0.623/0.613
ALOX15	ExcapeDB	1925/110264	AVALON	0.592/0.588

The final column shows the mean (repeated 3 times) balanced accuracy achieved for the best performing fingerprint across the calibration (80%) and test sets (20%). See also Figure 3.

^a^ https://pubchem.ncbi.nlm.nih.gov/bioassay/652039

^b^ https://pubchem.ncbi.nlm.nih.gov/bioassay/651820

^c^ https://pubchem.ncbi.nlm.nih.gov/bioassay/1527

^d^ https://pubchem.ncbi.nlm.nih.gov/bioassay/1063

^e^ https://pubchem.ncbi.nlm.nih.gov/bioassay/485364

^f^ https://pubchem.ncbi.nlm.nih.gov/bioassay/2642

^g^ https://pubchem.ncbi.nlm.nih.gov/bioassay/1430

^h^ https://pubchem.ncbi.nlm.nih.gov/bioassay/898

^i^ https://pubchem.ncbi.nlm.nih.gov/bioassay/504770

^j^ https://pubchem.ncbi.nlm.nih.gov/bioassay/540333

^k^ https://pubchem.ncbi.nlm.nih.gov/bioassay/360

**FIGURE 4:**
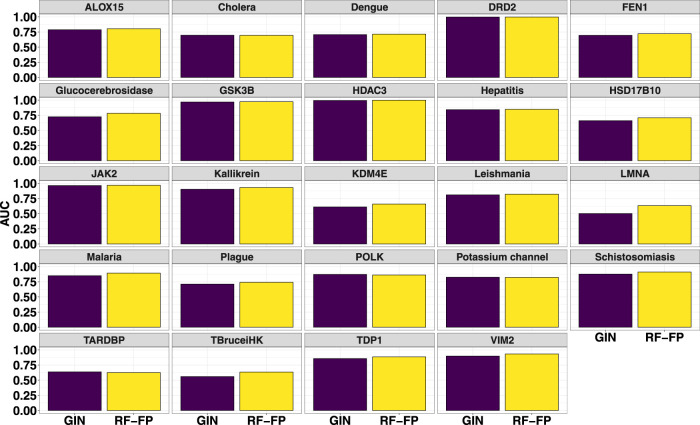
Comparison of random forest fingerprint models with graph isomorphism networks for the test sets (average of 3 random selections).

The fingerprint performance was compared with that of a graph isomorphism network (Xu et al., 2019; Wu et al., 2021b) (GIN) which is a powerful graph neural network (GNN) for graph classification (Kim and Ye, 2020). Using the torchdrug (Zhu et al., 2022) machine learning framework, the GIN was built with 4 hidden layers (number of hidden units set to 256), using an Adam optimizer and binary cross entropy loss function with batch normalization applied to every hidden layer. The model was subsequently trained for 100 epochs with the splits for train/valid/test sets set to 60%, 20% and 20% respectively. The barplots in Figure 4 show the comparison of the test set AUCs (mean of 3 independent runs) achieved by the RF and GNN models. As can be seen from the plots, for the majority of the data sets, RF models achieve relatively better metrics while for others the performances are comparable.

For all data sets, isolation forest (built using 500 trees) based outlier scores were calculated. Here, values closer to 1 indicate potential outliers while those around 0.50 typically suggest average outlierness. Values closer to 0 are more difficult to categorize. Supplementary Figure S5 in the SI shows the histograms of the distributions of the calculated scores. Examination of the plots show that for most of the data sets studied here, a cutoff of 0.5 (for some a lower value is recommended) may be used as a decision threshold to identify potential outliers (see Supplementary Figure S6 in the SI). Compared with other distance based approaches [such as the local outlier factor (Breunig et al., 2000) and one-class support vector machines (Chen et al., 2013)] where the algorithms typically try to fit the regions where the training data is the heavily concentrated, isolation forests do not use any distance metrics and instead rely on the concept that an ensemble of random trees are likely to produce shorter path lengths for outliers.

The model performance although encouraging for some does need significant improvement especially for data sets where the availability of actives is quite low. While a case for balanced data sets can be made, the skewed ratio between active and inactive compounds is a realistic representation of the high-throughput screening hit rates that are typically < 1% (Dreiman et al., 2021). For some data sets, improved performances were seen with substructure fingerprints such as AVALON that are based on pre-defined generic substructure patterns. For others, fingerprints such as ECFP/FCFP that take into account the neighborhood of each atom yielded slightly better classification models. Nonetheless, for many of the data sets (see Figure 3), the model metrics showed only marginal differences. In an earlier study, Riniker and Landrum (2013) observed strong correlations between the fingerprints which may explain the similarities in the obtained metrics. Overall, the choice of which fingerprint to use for modelling is far from trivial and is to a large extent dependent on the target. In this study, Avalon and FCFP4 fingerprints are generally seen standout as useful descriptors and may serve as useful starting points for future benchmarking studies. A potential avenue for improvement in prediction performance could be to combine 2D fingerprints with structure-based graph representations (Choo et al., 2023). Alternatively, one may look towards language representations which have recently been shown to yield good results on several classification and regression benchmarks (Ross et al., 2022).

### 3.3 Performance on regression tasks

**FIGURE 5:**
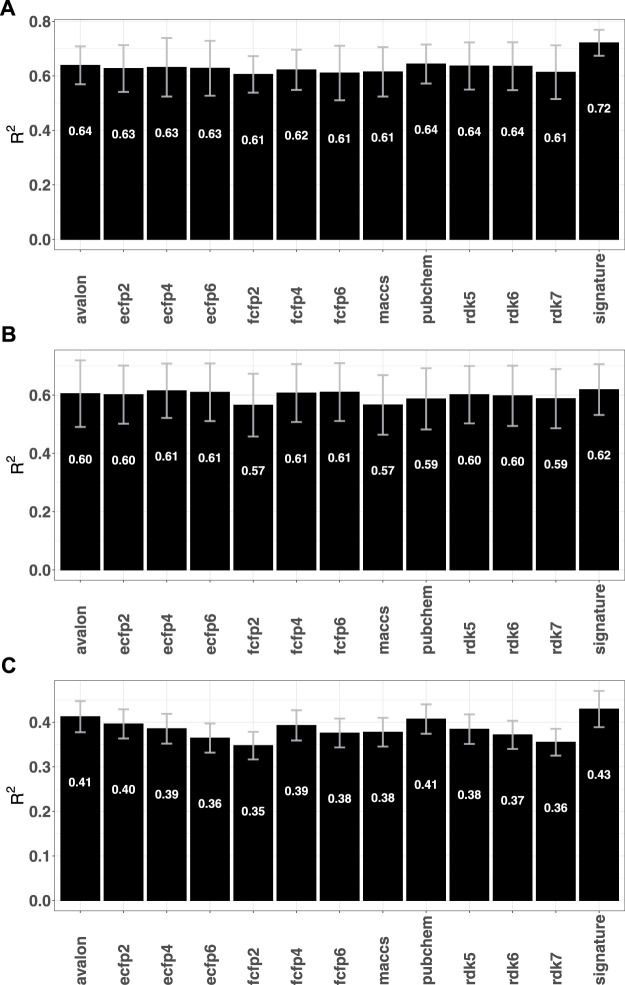
Mean *R* ^2^ obtained by the fingerprint models for different data sets **(A)** tuberculosis (Pires and Ascher, 2020) **(B)** GPCR (Velloso et al., 2021) and **(C)** Cancer Al-Jarf et al. (2021).

Given the relative success of the fingerprint-based RF classification models, an immediate question is whether the performances can be replicated for regression tasks. To this end, RF regression models were computed for a number of previously analysed data sets that used graph based signatures and other auxiliary attributes to identify potential candidates against *mycobacterium tuberculosis* (Pires and Ascher, 2020), cancer (Al-Jarf et al., 2021), and G protein-coupled receptors (Velloso et al., 2021) (GPCRs). A total of 1904 fingerprint-RF models were computed, spanning 36 different GPCRs, 8 organism-specific Mycobacteria species (*M. avium, M. caseum, M. kansasii, M. phlei, M. tuberculosis, M. bovis, M. fortuitum, M. smegmatis* and *M. intracellulare*) and 74 distinct cancer cell lines corresponding to 9 tumor types (renal, breast, CNS, colon, leukemia melanoma, non small cell lung, ovarian, prostate, and small cell lung). Supplementary Figures S7–S9 in the SI summarize the regression performances of the different fingerprints. When compared with the graph signature based approaches, although marginal improvements were seen for some cases, the overall performance measured in terms of the squared Pearson correlation (*R* ^2^) was largely found to be comparable, with only models for tuberculosis yielding slightly lower *R* ^2^ values (see Figure 5). The fingerprint performance observed for these data sets mirrors the trends seen for a number of ADMET-related responses that were studied in a previous article [see (Venkatraman, 2021)] and suggest that purely fingerprint-based models may have low predictive utility for regression.

## 4 Software implementation and usage

**FIGURE 6:**
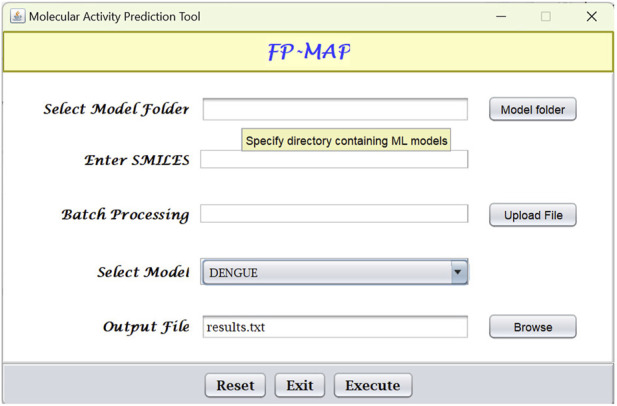
Graphical user interface for FPMAP. Users can upload a SMILES file (“Batch processing”) or alternatively enter a single SMILES string for evaluation. Prediction results are written to the output file specified.

Fingerprint calculations were carried out using the CDK (Willighagen et al., 2017) and RDKit (Landrum, 2022) libraries. Random forests models were built using the R (R Core Team, 2022) package ranger (Wright and Ziegler, 2017). The models were subsequently converted to predictive model markup language (PMML) which is an XML format that facilitates sharing of models between PMML compliant applications. For ease-of-use, a Java-based graphical user interface (see Figure 6) has been created which integrates the Java Evaluator API (https://github.com/jpmml) for model evaluation. In addition to the GUI, FP-MAP has also been made available as a command line interface.

## 5 Conclusion

This article sets out to assemble a comprehensive catalogue of predictive models for small molecules with potential bioactivity against various targets and diseases. Previous studies have provided only fragments of the large spectrum of molecule pharmacodynamics and bioactivity prediction models, many of which are not easily accessible. Encouraged by the initial predictive performance of the fingerprints on over 80 targets for which close to 1,000 models were computed, machine learning algorithms were applied to a number of important targets for which freely accessible prediction models are not available (to the best of the author’s knowledge). For the 24 data sets included in the current release of the software, the fingerprint-based binary classification performances for severely imbalanced datasets ranged from moderate (AUC ≈0.61) to high (AUC >0.90) and outperform alternative approaches. FP-MAP provides a simple and easy to use platform for predicting activity of novel compounds as well as for benchmarking studies. As more and more curated data sets emerge (Béquignon et al., 2023; Buterez et al., 2023), future efforts will focus on expanding the palette of targets and diseases.

## Data Availability

Publicly available datasets were analyzed in this study. This data can be found here: https://doi.org/10.5281/zenodo.7983590.
