# Supplementary material for: FP-MAP: an extensive library of fingerprint-based molecular activity prediction tools
Source: Front Chem. 2023 Aug 15;11:1239467. doi: 10.3389/fchem.2023.1239467 (PMC10462816; doi:10.3389/fchem.2023.1239467)
Supplement: Supplementary file 1 [file DataSheet1.PDF]

Supplementary Material

1 SUPPLEMENTARY TABLES AND FIGURES

1.1 Tables

Table S1: Summary of the data sets used for classification models. Data for the models was taken from multiple literature articles: breast cancer (He et al., 2021), cardiac toxicity (Iftkhar et al., 2022), chemical toxicology (Allen et al., 2020).

Target/Cell line	Actives/Inactives	Target/Cell line	Actives/Inactives
Breast Cancer			
Bcap37	110/165	BT-20	121/171
BT-474	640/171	BT-549	798/384
HBL-100	183/133	HS-578T	339/130
MCF-7	16310/13068	MDA-MB-231	7136/4066
MDA-MB-361	246/121	MDA-MB-435	2361/669
MDA-MB-453	225/215	MDA-MB-468	1522/464
SK-BR-3	1522/504	T-47D	2199/936
Cardiac toxicity			
Arhhythmia	706/862	Cardiac failure	307/846
Heart blockage	466/466	hERG toxicity	6788/6035
Hypertension	705/669	Myocardial	401/402
Chemical Toxicology			
AChE	2611/1964	ADORA2A	3943/2082
ADRA2A	842/1013	AR	2637/7283
ADRB1	1260/1080	ADRB2	1943/2012
OPRD1	3006/1219	DRD1	1350/1990
DRD2	5694/1136	SLC6A3	2509/1916
EDNRA	1285/1150	NR3C1	3018/6972
KCNH2	4895/3245	HRH1	1275/1105
OPRM1	3610/2305	CHRM1	2014/1241
CHRM2	1633/2032	CHRM3	1537/1113
SLC6A2	2910/1940	HTR2A	3757/1033
HTR3A	451/1054	SLC6A4	4041/1134
LCK	1732/523	AVPR1A	619/1056
AGTR1	806/1179	AKT1	2765/1220
BACE1	6016/2604	BCHE	1400/2145
CASP1	1369/3196	CASP3	1177/1828
CASP8	330/1130	CHRM5	679/1081
CHUK	316/1069	CSF1R	1336/1049

Continued on next page

Table S1 – continued from previous page

Target	Actives/Inactives	Target	Actives/Inactives
CSNK1D	708/1027	EDNRB	809/1236
ELANE	2134/1371	EPHA2	528/1102
FGFR1	2163/1207	FKBP1A	354/1006
FLT1	1088/2077	FLT4	674/1081
FYN	420/1075	GSK3B	2549/1256
HDAC3	1051/1139	IGF1R	2483/1132
INSR	887/1093	KDR	7816/1579
LTB4R	350/1030	LYN	454/1046
MAPK1	6209/11076	MAPK9	1227/1088
MAPKAPK2	829/1156	MET	2871/1144
MMP13	2388/1112	MMP2	2938/1677
MMP3	1759/1036	MMP9	2582/1848
NEK2	298/1057	P2RY1	560/1100
PAK4	380/1100	PDE4A	653/1017
PDE5A	1551/1174	PIK3CA	4724/2086
PPARG	4362/7283	PTPN1	1471/2179
PTPN11	354/1211	PTPN2	339/1206
RAF1	1351/1084	RARA	356/3249
RARB	298/3347	ROCK1	1293/1117
RPS6KA5	224/1036	SIRT2	361/1284
SIRT3	151/1074	SRC	2704/1531
TACR2	876/1914	TBXA2R	978/1922
TEK	788/1132		

Table S2: Summary of the data sets used for regression models. Data for the models was taken from multiple literature articles: cancer (Al-Jarf et al., 2021), GPCR (Velloso et al., 2021), tuberculosis (Pires and Ascher, 2020).

Target/Cell line	#Compounds	Target/Cell line	#Compounds
	GPCR		
P08173	978	P08908	3790
P08912	959	P0DMS8	3513
P20309	2008	P21452	922
P21917	2335	P24530	987
P28335	3118	P29275	2109
P30542	3833	P30968	1373
P34995	741	P35346	747
P35348	1898	P35372	5275
P41180	535	P46663	756
P47871	1006	P47900	568
P48039	1043	P50406	3044
P51677	1131	Q14416	1168
Q14833	579	Q16602	757
Q8TDS4	504	Q8TDU6	443
Q96LB2	93	Q99500	1088
Q99705	3721	Q99835	718
Q9H228	417	Q9HC97	480
Q9Y5N1	3597	Q9Y5Y4	2749
	Cancer		
breast_BT_549	6595	breast_HS_578T	6890
breast_MCF7	7453	breast_MDA_MB_231_ATCC	6990
breast_MDA_MB_468	1941	breast_T_47D	6825
CNS_SF_268	14241	CNS_SF_295	14473
CNS_SF_539	13865	CNS_SNB_19	14365
CNS_SNB_75	13820	CNS_SNB_78	8190
CNS_U251	14569	CNS_XF_498	6977
colon_COLO_205	14067	colon_DLD_1	8254
colon_HCC_2998	12809	colon_HCT_116	14090
colon_HCT_15	14044	colon_HT29	14483
colon_KM12	14291	colon_KM20L2	7984
colon_SW_620	14668	leukemia_CCRF_CEM	13558
leukemia_HL_60TB	13400	leukemia_K_562	14147
leukemia_MOLT_4	13987	leukemia_P388_ADR	881
leukemia_P388	895	leukemia_RPMI_8226	13383
leukemia_SR	10992	melanoma_LOX_IMVI	13617
melanoma_M14	14008	melanoma_M19_MEL	8448
melanoma_MALME_3M	13778	melanoma_MDA_MB_435	7001

Continued on next page

Table S2 – continued from previous page

#Compounds	Target/Cell line	#Compounds	
melanoma_MDA_N	4940	melanoma_SK_MEL_28	14350
melanoma_SK_MEL_2	13591	melanoma_SK_MEL_5	13913
melanoma_UACC_257	14270	melanoma_UACC_62	14371
NSCL_A549_ATCC	14639	NSCL_EKVX	13919
NSCL_HOP_18	6836	NSCL_HOP_62	13883
NSCL_HOP_92	12973	NSCL_LXFL_529	7629
NSCL_NCI_H226	13107	NSCL_NCI_H23	14387
NSCL_NCI_H322M	14218	NSCL_NCI_H460	14243
NSCL_NCI_H522	13182	ovarian_IGROV1	14389
ovarian_NCI_ADR_RES	7458	ovarian_OVCAR_3	14207
ovarian_OVCAR_4	13878	ovarian_OVCAR_5	14010
ovarian_OVCAR_8	14604	ovarian_SK_OV_3	13818
prostate_DU_145	7016	prostate_PC_3	7017
renal_786_0	13873	renal_A498	13057
renal_ACHN	14006	renal_CAKI_1	13467
renal_RXF_393	13199	renal_RXF_631	7054
renal_SN12C	14554	renal_SN12K1	907
renal_TK_10	13837	renal_UO_31	14353
SCL_DMS_114	8385	SCL_DMS_273	7827
	Tuberculosis		
avium	1119	bovis	290
caseum	254	fortuitum	571
general	15766	intracellulare	368
kansasii	1000	phlei	230
smegmatis	2115	tuberculosis	13991

1.2 Figures

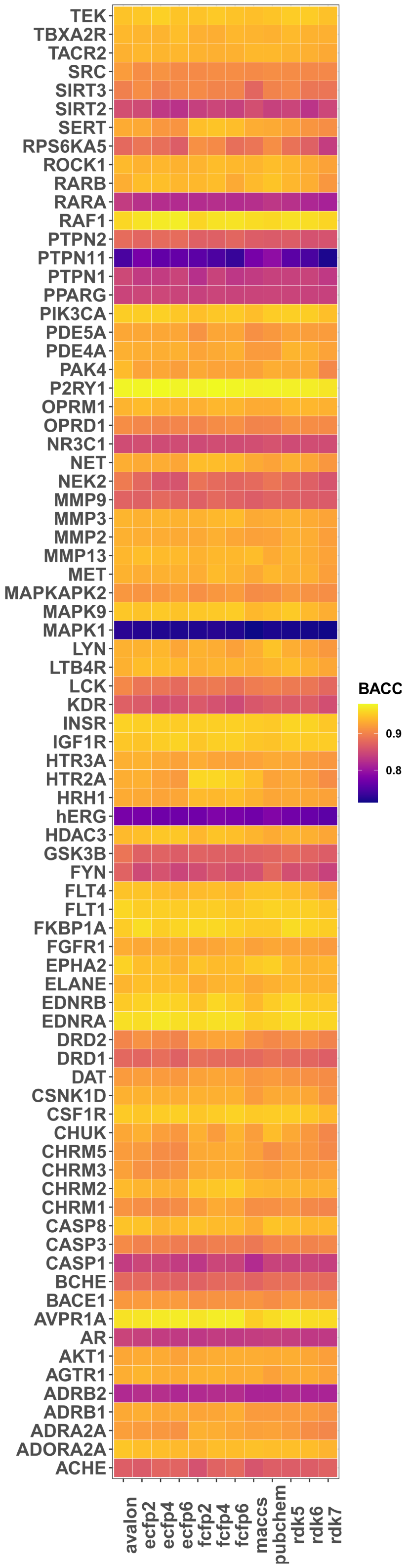

Figure S1. Heatmap showing the distribution of the cross-validated balanced accuracies on the chemical toxicity data sets (Allen et al., 2020) obtained by the random forests-based fingerprint models. Values reported are the mean of 3 independent runs.

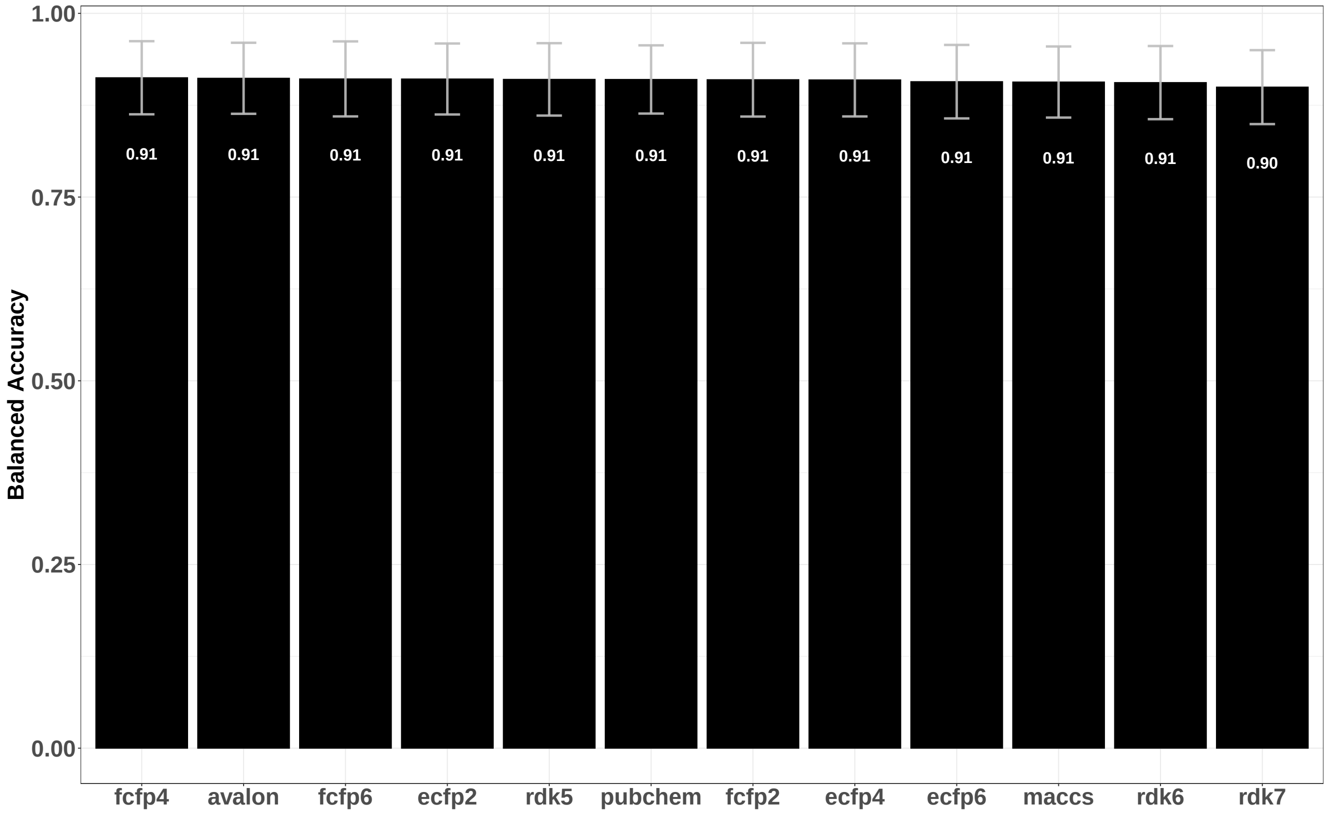

Figure S2. Barplot showing the mean balanced accuracies (averaged over 79 chemical toxicity targets) obtained by the random forests-based fingerprint models (Allen et al., 2020).

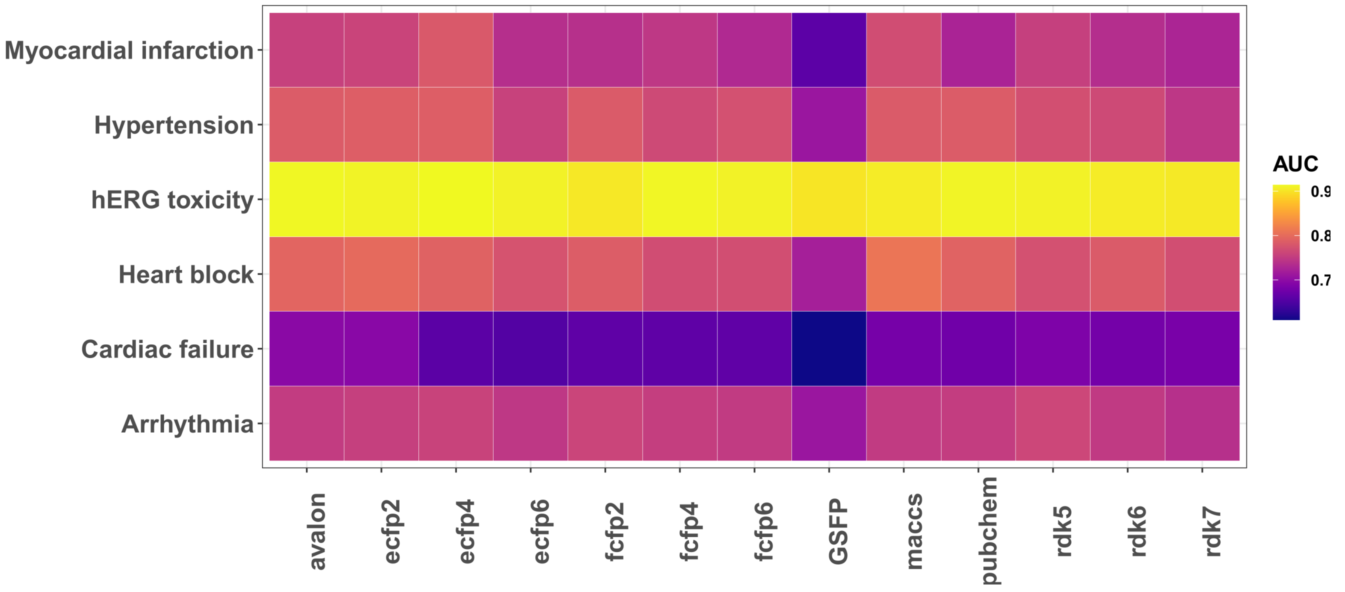

Figure S3. Figure shows the 5-fold cross-validated AUCs for the fingerprint models for different cardio toxicity outcomes. The values are compared 5-fold cross-validated AUCs for models that used a combination of graph signatures and fingerprints as descriptors (see Iftkhar et al. (2022)).

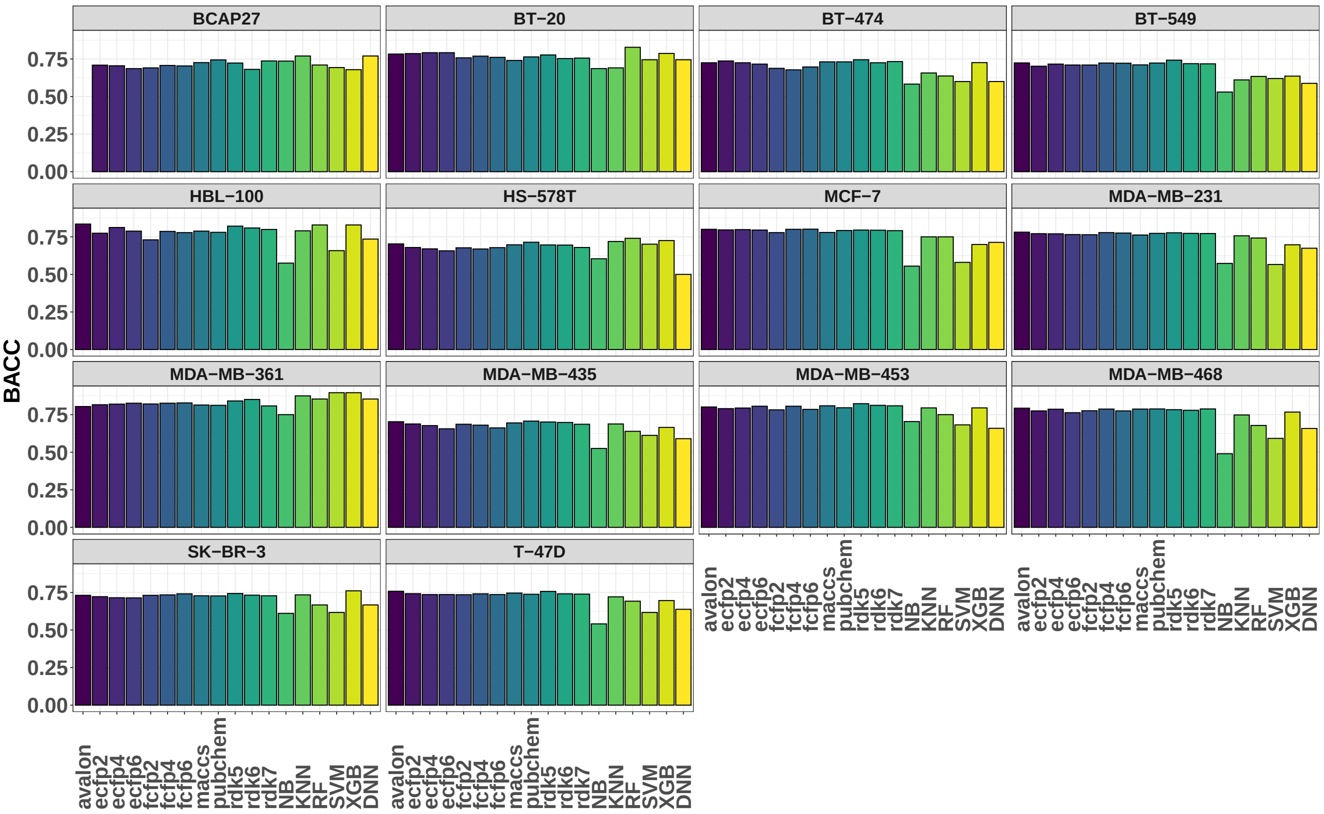

Figure S4. Figure shows the 5-fold cross-validated balanced accuracies (BACC) for the fingerprint models obtained for breast cancer outcomes. The values are compared with 10-fold cross-validated BACC for descriptor-based ML models: random forests (RF), XGBoost (XGB), deep neural networks (DNN), naive Bayes (NB), k-nearest neighbours (KNN) and support vector machines (SVM). Values for the 6 additional ML approaches were taken from He et al. (2021).

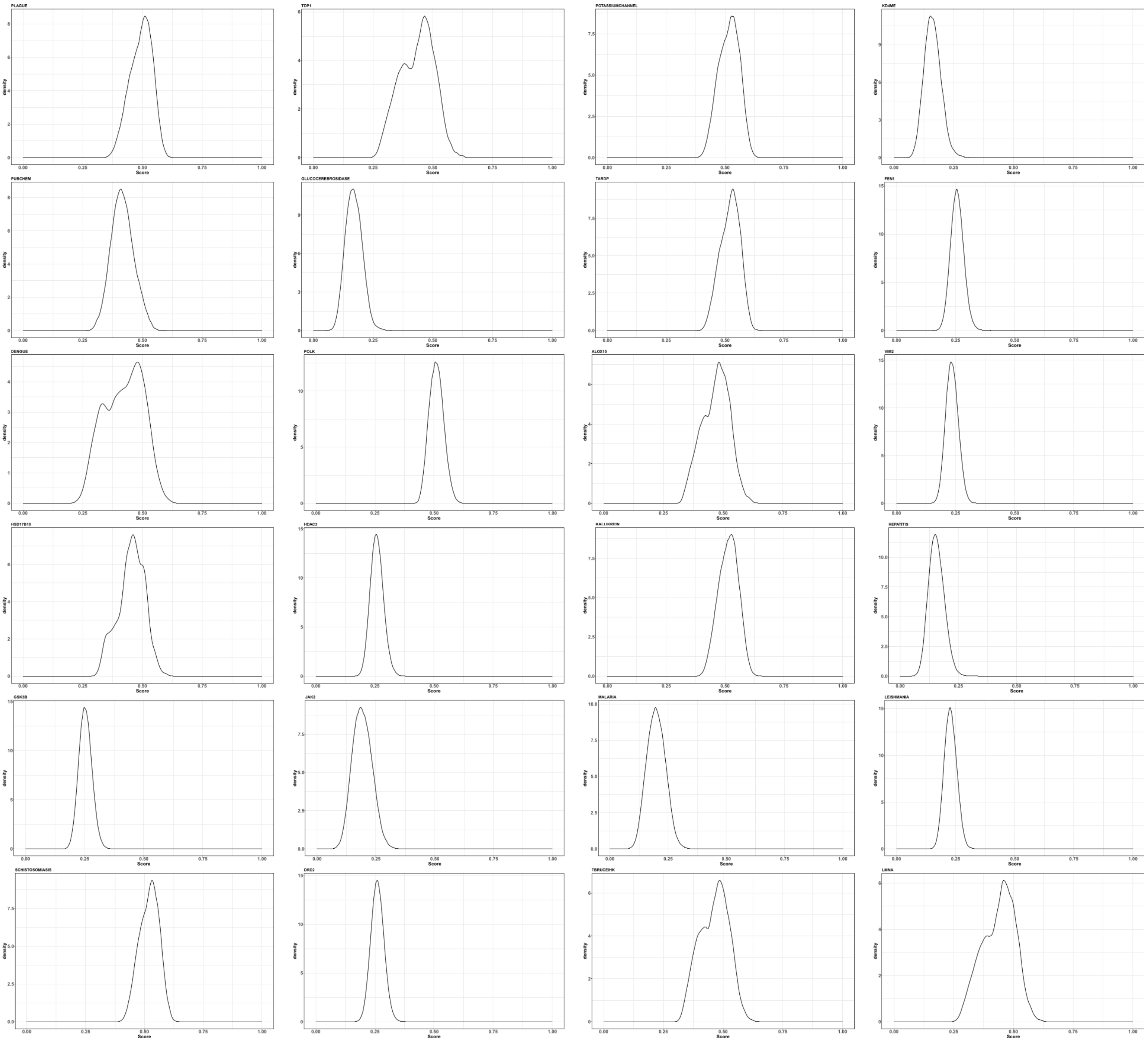

Figure S5. Density plot of the isoforest scores calculated for each observation in the data set. Higher scores imply more outlierness.

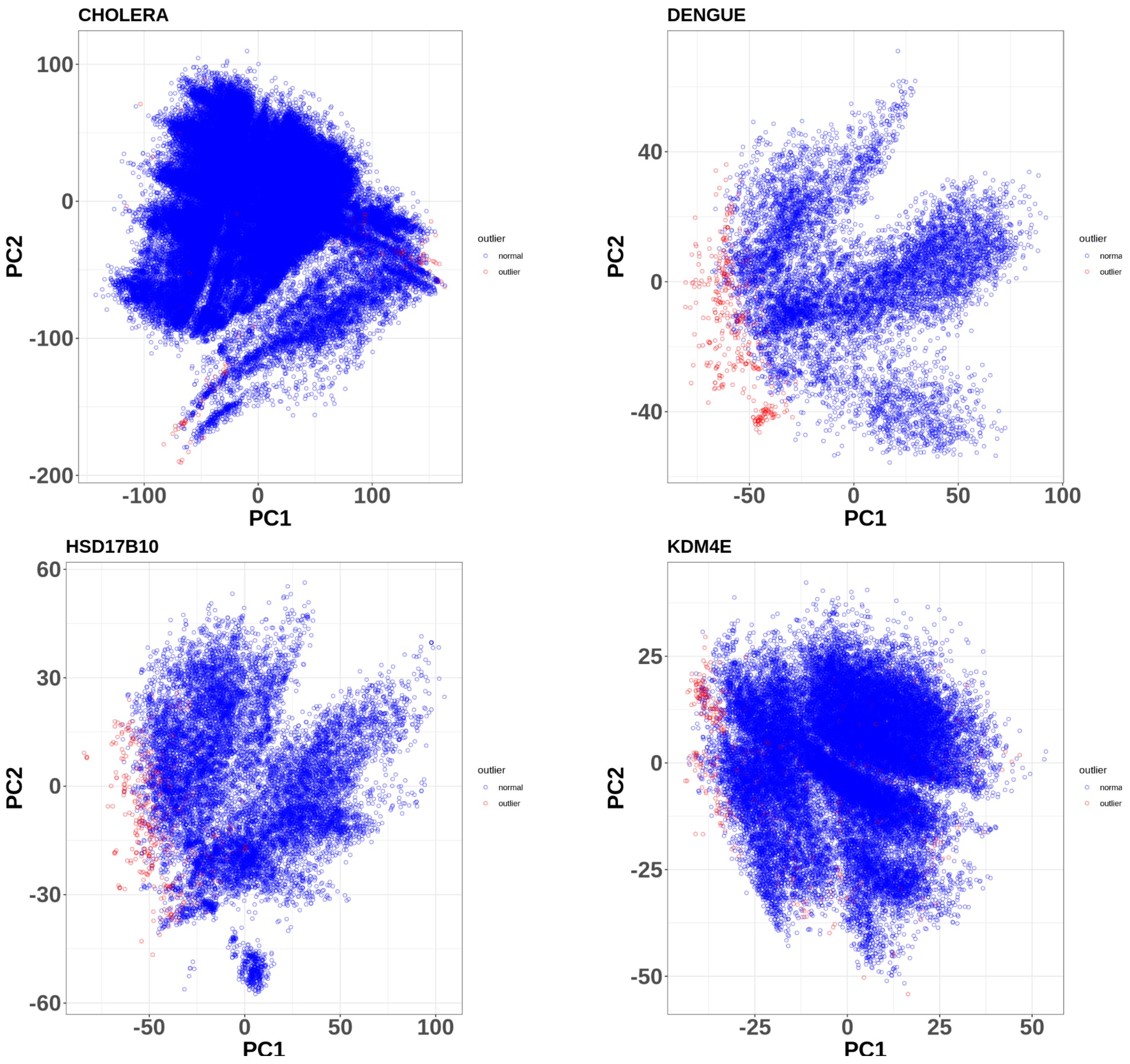

Figure S6. A logistic principal component analysis (Landgraf and Lee, 2020) was carried out on the fingerprints using the R package `logisticpca` (Landgraf and Lee, 2015) for 4 different data sets: cholera, dengue, HSD17B19 and KDM4E. Using the outlier scores calculated by the isoforest algorithm, data points were marked as outliers (shown in red) based on cutoffs of 0.55, 0.55, 0.25, 0.55 for dengue, HSD17B10, KDM4E and Cholera respectively. The outliers are seen to typically lie on the periphery and can be indicative of out of domain compounds.

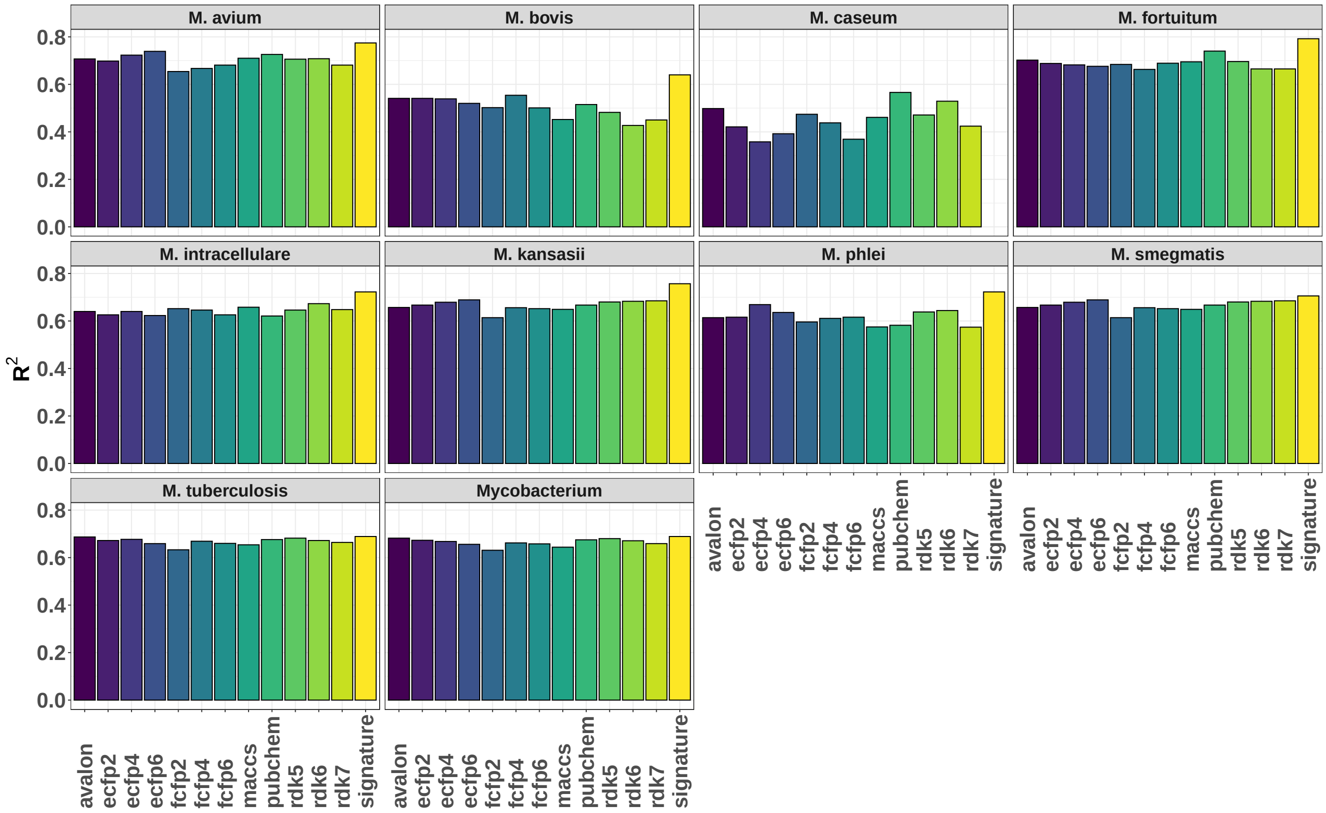

Figure S7. For the tuberculosis strains, figure shows a comparison of the 5-fold cross-validated R^2 for the fingerprint models with values obtained graph signature-based descriptor models (reported for 10-fold cross-validation). See Pires and Ascher (2020).

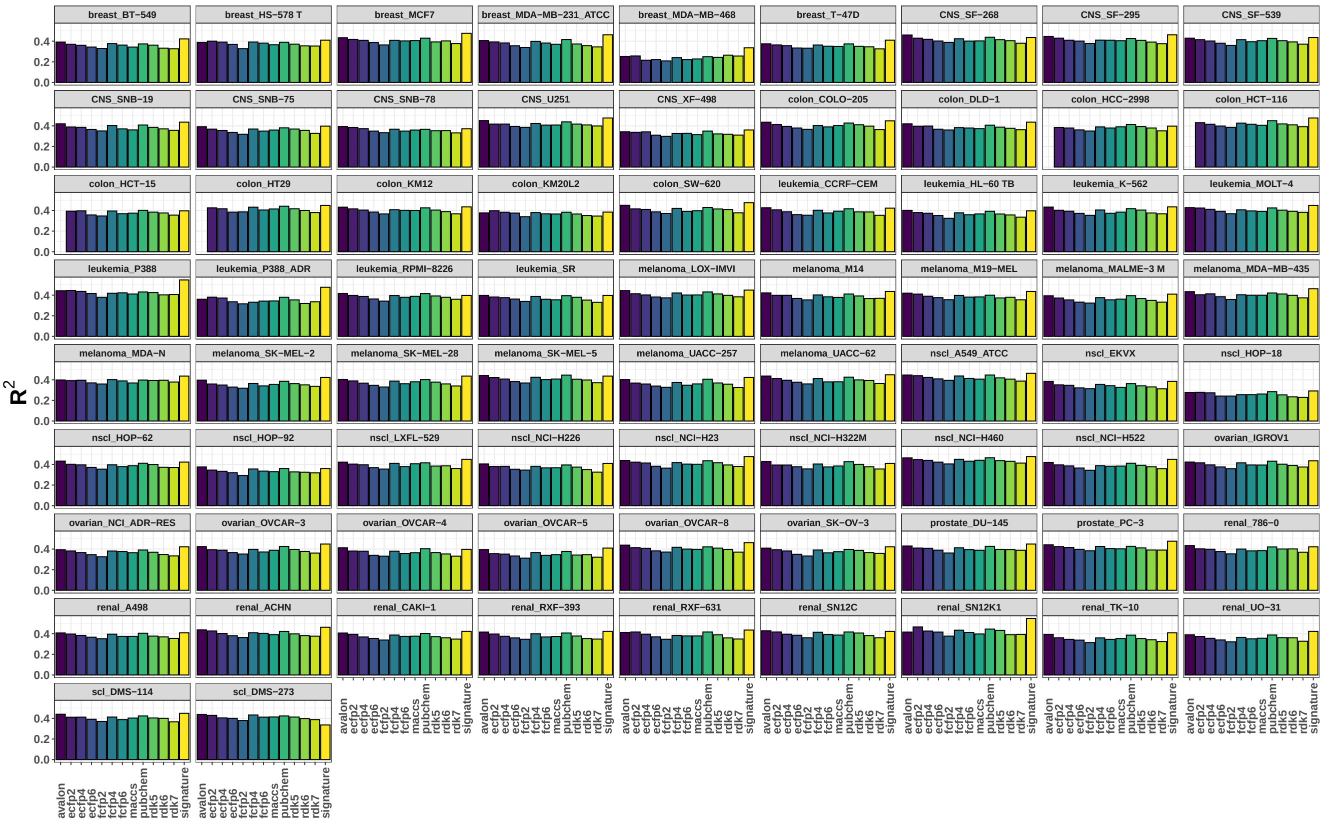

Figure S8. Figure shows a comparison of the 5-fold cross-validated R^2 for the fingerprint models (created for cancer cell lines) with values obtained graph signature-based descriptor models (reported for 10-fold cross-validation). See Al-Jarf et al. (2021)

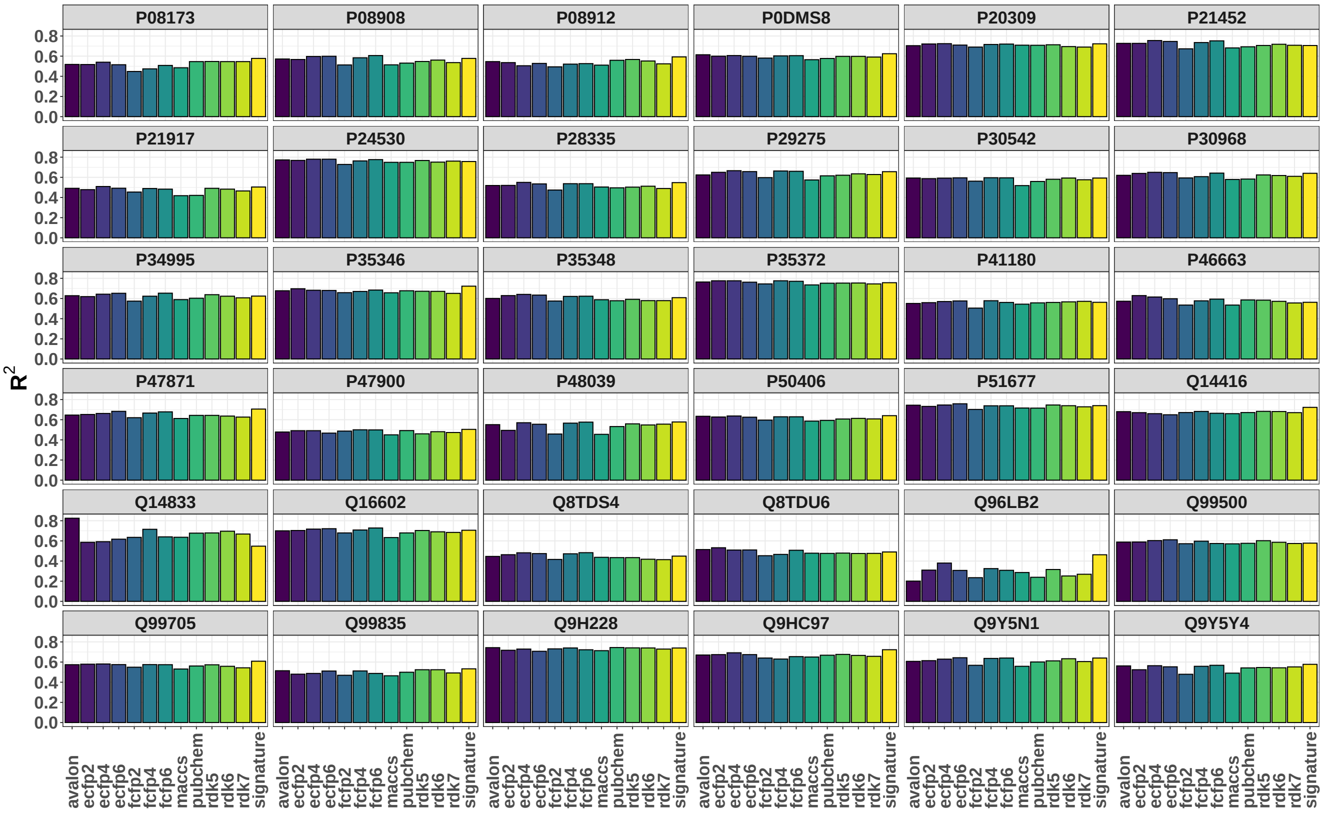

Figure S9. Figure shows a comparison of the 5-fold cross-validated R^2 for the fingerprint models (created for GPCRs) with values obtained graph signature-based descriptor models (reported for 10-fold cross-validation). See Velloso et al. (2021).

REFERENCES

Al-Jarf, R., de Sá, A. G. C., Pires, D. E. V., and Ascher, D. B. (2021). pdCSM-cancer: Using graph-based signatures to identify small molecules with anticancer properties. *J Chem. Inf. Model.* 61, 3314–3322. doi:10.1021/acs.jcim.1c00168

Allen, T. E. H., Wedlake, A. J., Gelžinytė, E., Gong, C., Goodman, J. M., Gutsell, S., et al. (2020). Neural network activation similarity: a new measure to assist decision making in chemical toxicology. *Chem. Sci.* 11, 7335–7348. doi:10.1039/d0sc01637c

He, S., Zhao, D., Ling, Y., Cai, H., Cai, Y., Zhang, J., et al. (2021). Machine learning enables accurate and rapid prediction of active molecules against breast cancer cells. *Front. Pharmacol.* 12. doi:10.3389/fphar.2021.796534

Iftkhar, S., de Sá, A. G. C., Velloso, J. P. L., Aljarf, R., Pires, D. E. V., and Ascher, D. B. (2022). cardiotoxcsm: A web server for predicting cardiotoxicity of small molecules. *J Chem. Inf. Model.* 62, 4827–4836. doi:10.1021/acs.jcim.2c00822

Landgraf, A. J. and Lee, Y. (2015). *Dimensionality Reduction for Binary Data through the Projection of Natural Parameters*. Tech. Rep. 890, Department of Statistics, The Ohio State University

Landgraf, A. J. and Lee, Y. (2020). Dimensionality reduction for binary data through the projection of natural parameters. *Journal of Multivariate Analysis* 180, 104668. doi:10.1016/j.jmva.2020.104668

Pires, D. E. V. and Ascher, D. B. (2020). mycoCSM: Using graph-based signatures to identify safe potent hits against mycobacteria. *J Chem. Inf. Model.* 60, 3450–3456. doi:10.1021/acs.jcim.0c00362

Velloso, J. P. L., Ascher, D. B., and Pires, D. E. V. (2021). pdCSM-GPCR: predicting potent GPCR ligands with graph-based signatures. *Bioinform. Adv.* 1. doi:10.1093/bioadv/vbab031
